# Analysis of the Storage Stability Property of Carbon Nanotube/Recycled Polyethylene-Modified Asphalt Using Molecular Dynamics Simulations

**DOI:** 10.3390/polym13101658

**Published:** 2021-05-20

**Authors:** Caihua Yu, Kui Hu, Qilin Yang, Dandan Wang, Wengang Zhang, Guixiang Chen, Chileshe Kapyelata

**Affiliations:** 1 College of Civil Engineering, Henan University of Technology, Zhengzhou 450001, China; enyue0919@gmail.com (C.Y.); mailwangdandan0919@gmail.com (D.W.); chileshekapyelata21@gmail.com (C.K.); 2 School of Transportation Science and Engineering, Harbin Institute of Technology, Harbin 150090, China; yangqilin@stu.hit.edu.cn; 3 School of Civil and Architectural Engineering, Shandong University of Technology, Zibo 255049, China; ziwuzizwg@sdut.edu.cn

**Keywords:** recycled polyethylene (RPE), modified asphalt, carbon nanotubes (CNT), molecular dynamics (MD) simulation, storage stability, interaction mechanisms

## Abstract

Carbon nanotubes (CNTs) can improve the storage properties of modified asphalt by enhancing the interfacial adhesion of recycled polyethylene (RPE) and base asphalt. In this study, the interaction of CNT/RPE asphalt was investigated using molecular dynamics simulation. The base asphalt was examined using a 12-component molecular model and verified by assessing the following properties: its four-component content, elemental contents, radial distribution function (RDF) and glass transition temperature. Then, the adhesion properties at the interface of the CNT/RPE-modified asphalt molecules were studied by measuring binding energy. The molecular structural stability of CNTs at the interface between RPE and asphalt molecules was analyzed through the relative concentration distribution. The motion of molecules in the modified asphalt was studied in terms of the mean square displacement (MSD) and diffusion coefficient. The results showed that CNTs improved the binding energy between RPE and base asphalt. CNTs not only weakened the repulsion of RPE with asphaltenes and resins, but also promoted the interaction of RPE with light components, which facilitated the compatibility of RPE with the base asphalt. The change in the interaction affected the molecular motion, and the molecular diffusion coefficient in the CNT/RPE-modified asphalt system was significantly smaller than that of RPE-modified asphalt. Moreover, the distribution of the asphaltene component was promoted by CNTs, resulting in the enhancement of the storage stability of RPE-modified asphalt. The property indexes indicated that the storage stability was significantly improved by CNTs, and better viscoelastic properties were also observed. Our research provides a foundation for the application of RPE in pavement engineering.

## 1. Introduction

Currently, humans have produced more than 8.3 billion tons of virgin plastics and more than 6300 metric tons of plastic waste, the vast majority of which goes into nature and poses a serious threat to the environment [1]. The recycling of waste plastics is a global issue. Road engineering has become an effective way to consume and reuse plastic waste due to its significant advantages, such as high consumption and low energy consumption [2]. The types and composition of plastics are complex, consisting mainly of high-density polyethylene (HPPE), medium-density polyethylene (MDPE), low-density polyethylene (LDPE), polypropylene (PP), polystyrene (PS), polyvinyl chloride (PVC), polyethylene terephthalate (PET) and polyurethane resins, with the largest production of PE-based plastics [3].

PE as a modifier has been shown to improve the high-temperature performance, low-temperature performance and fatigue resistance of asphalt [4]. PE provides enhancements to the thermodynamic, chemical and physical properties of the asphalt [5]. Moreover, the properties of modified asphalt are strongly related to the molecular structure of PE, with MDPE-modified asphalt showing the best rutting resistance, whereas the highly branched PE-modified asphalt has shown better low-temperature performance [6]. In addition, PE-modified asphalt has shown better adhesion, elasticity and hardness relative to matrix asphalt [7,8]. PE-modified asphalt has attracted a lot of attention because it can improve the performance of the asphalt matrix and help eliminate “white pollution”. However, the weak interface between PE and the asphalt matrix causes serious storage stability problems in the production and storage of PE-modified asphalt [9,10].

To improve the storage stability of PE-modified asphalt, many additives have been employed [11,12], the most popular of which is carbon nanotubes (CNTs) [13,14]. A CNT, a carbon nanomaterial of wide interest, is a tubular one-dimensional (1D) material com-posed of carbon atoms with excellent thermodynamic properties, mechanical strength and specific surface area [15,16]. When CNTs are added to the PE-modified asphalt matrix, their special pull-out effect has a significant strengthening effect on the weak interface between PE and asphalt, which, in turn, improves the storage stability and high-temperature performance of the modified asphalt [17,18]. Previous studies have focused on the enhancement effect of CNT on PE-modified asphalt [14]; however, the microscopic mechanism underlying this phenomenon is not well understood, which is not conducive to the directional design of modified asphalt properties.

Many techniques have been used for the exploration of asphalt modification mechanisms. Micro-scale asphalt morphology can be investigated by means of atomic force microscopy (AFM), the interface characteristics of the modifier and asphalt matrix can be obtained using scanning electron microscopy (SEM), fluorescence microscopy can be used to observe the biphasic structure of modified asphalt and Fourier infrared spectroscopy (FTIR) can be used to determine the group changes in the modified asphalt system [19,20]. Although these techniques can reflect the microscopic state of asphalt to some extent, most of them are only phenomenal or qualitative descriptions, which can hardly reveal the microscopic mechanism of macroscopic phenomena clearly [21].

Molecular dynamics simulation is an excellent tool for the quantitative characterization of the micro-state of materials, and the basis for its use is the construction of molecular models. For extremely complex mixtures like asphalt, it is impossible, and at the same time unnecessary, to construct a realistic molecular model. The most efficient way is to construct a model based on the asphalt component method, using the average molecular method [22] or the assembly method [23]. There have been many reports using molecular dynamics simulations to explore the quantitative relationship between the molecular content and macroscopic properties of asphalt, which involve numerous properties of asphalt, such as glass transition temperature [24], self-healing behavior [25,26], diffusion behavior [27] and aging behavior [28]. Currently, modified asphalt has been widely used in road engineering and molecular dynamics simulations have been used to study modified asphalt, which involves the interaction between components [29], contact angle [30], various mechanical properties [31], molecular agglomeration and structure [32], etc. Therefore, it is feasible to use molecular dynamics simulations to explore the microscopic mechanisms of macroscopic phenomena.

The objective of this study was to explore the microscopic mechanism of the storage stability enhancement of CNT/RPE-modified asphalt using molecular dynamics simulations. CNT/RPE-modified asphalt samples were prepared, and the changes in their properties before and after CNT addition were measured. Molecular models of virgin asphalt, RPE-modified asphalt and CNT/RPE-modified asphalt were developed, and the rationality of the asphalt models was verified based on the four-component content, radial distribution function (RDF), elemental content and molecular structure. The binding energy was used to characterize the strength of interfacial interaction, the relative concentration distribution was used to characterize the molecular structure and the diffusion coefficient was used to characterize the motion of the molecules in the system.

## 2. Methodology

### 2.1. Preparation of CNT/RPE-Modified Asphalt

In this study, ESSO asphalt (Mobil, TX, USA) with a property grade of 90 was selected as the base asphalt, and the basic properties are shown in Table 1. RPE bags (the main component was low-density polyethylene (LDPE)) were provided by Zhengzhou Green Source Waste Recycling Co. and extruded into granular form using a double screw rod. In addition, CNT was purchased from Shanghai MACKLIN Company under product number C805971, which consisted of single-walled carbon nanotubes with an outer diameter of 1–2 nm and a length of 20 μm.

**Figure 1:**
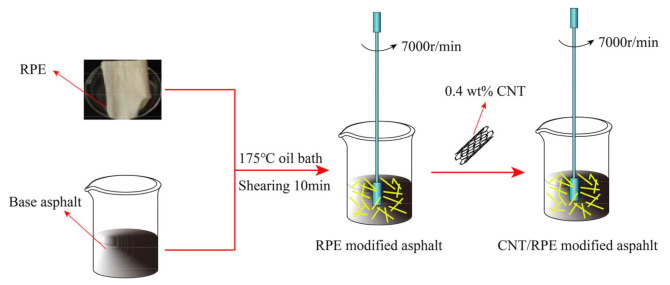
Sample preparation process of RPE-modified asphalt and CNT/RPE-modified asphalt.

The preparation processes of the RPE-modified asphalt and CNT/RPE-modified asphalt samples used in this study are shown in Figure 1. The CNT/RPE-modified asphalt samples were prepared using a high-speed shear with the content of RPE set at 5% (mass fraction, mass ratio of RPE to base asphalt) and the content of CNT set at 0.4% (mass ratio of CNT to RPE-modified asphalt).

### 2.2. Perperty Tests

The high-temperature rheological properties of RPE-modified asphalt and CNT/RPE-modified asphalt were measured using a dynamic shear rheometer (DSR, Anton Paar GmbH in Graz, Austria) according to AASHTO T 315. The effect of CNT on the storage stability of RPE-modified asphalt was characterized through test tube experiments, and the test standard was ASTM D7173. The effect of CNT on the low-temperature properties of RPE-modified asphalt was characterized in terms of 10 °C ductility, and the test standard was ASTM D113.

### 2.3. Model Building

#### 2.3.1. Asphalt Molecular Models

**Figure 2:**
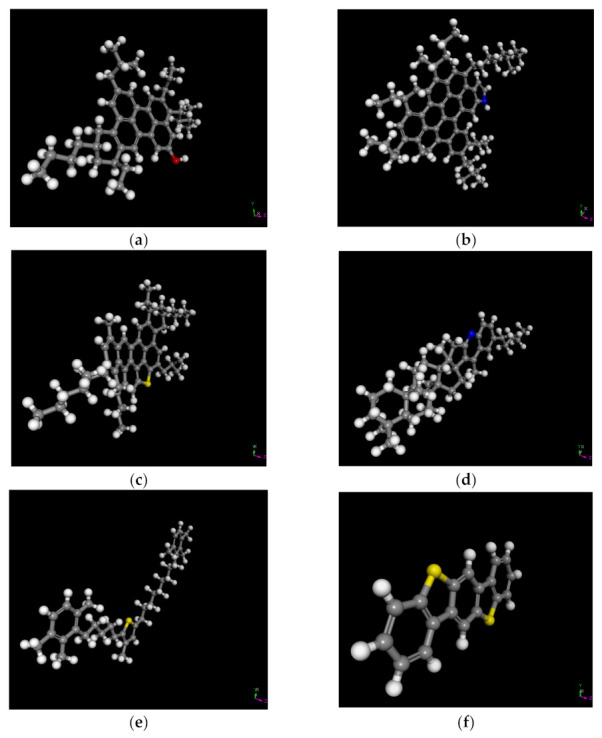

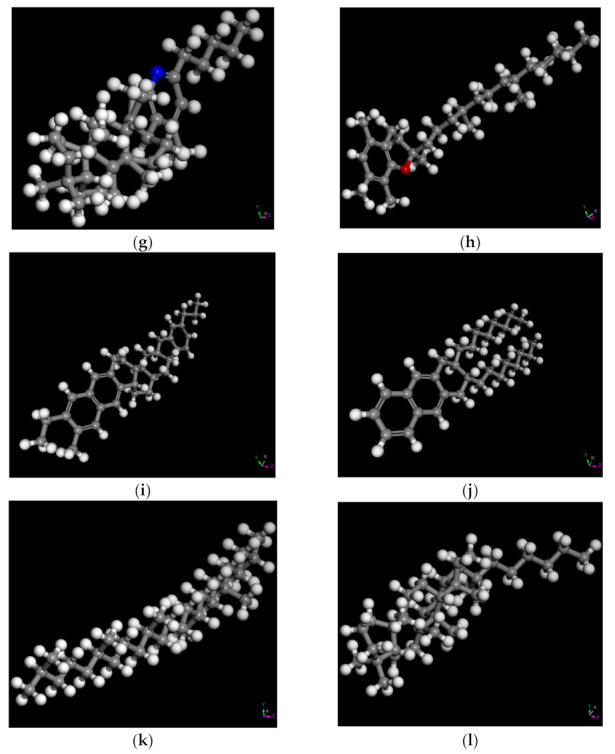
Molecular models of base asphalt. (**a**) Asphaltene A, (**b**) Asphaltene B, (**c**) Asphaltene C, (**d**) Resin A, (**e**) Resin B, (**f**) Resin C, (**g**) Resin D, (**h**) Resin E, (**i**) Aromatic A, (**j**) Aromatic B, (**k**) Saturate A, (**l**) Saturate B.

The molecular models of asphalt used in this study were the 12 molecular models proposed in the literature [22], and these molecular models are shown in Figure 2.

**Figure 3:**
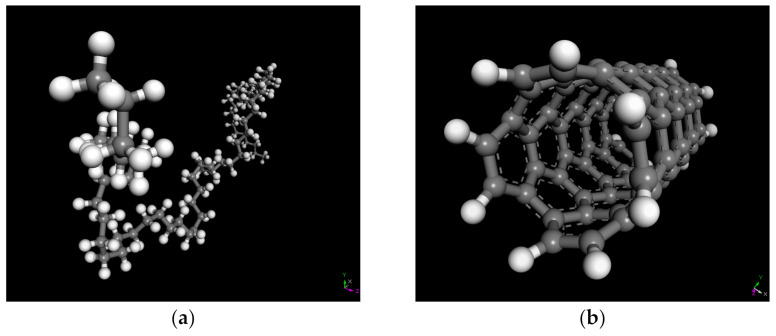
Molecular models of PE (**a**) and CNT (**b**).

It has to be mentioned that the RPE molecule is consistent with the PE molecule. For molecular dynamics simulations, the choice of polymer polymerization degree has an important effect on both simulation accuracy and simulation cost. In order to minimize the simulation time required, while maintaining the simulation accuracy, the optimal PE polymerization degree needs to be chosen. Previous studies by the authors have shown that the optimal degree of polymerization of PE is 35 [20]; therefore, the PE polymerization degree used in this study was 35. The PE molecular model used in this study is shown in Figure 3a.

The molecular model of a CNT varies with diameter and length, and a CNT with a diameter of 5.42 Å and a length of 12.30 Å was used in this study. It has to be mentioned that hydrogen atoms were added to the unsaturated carbon atoms in order to eliminate the unsaturated boundary effect of CNTs. The CNT molecular model is shown in Figure 3b.

#### 2.3.2. Modified Asphalt Models and Molecular Dynamic Simulation

**Figure 4:**
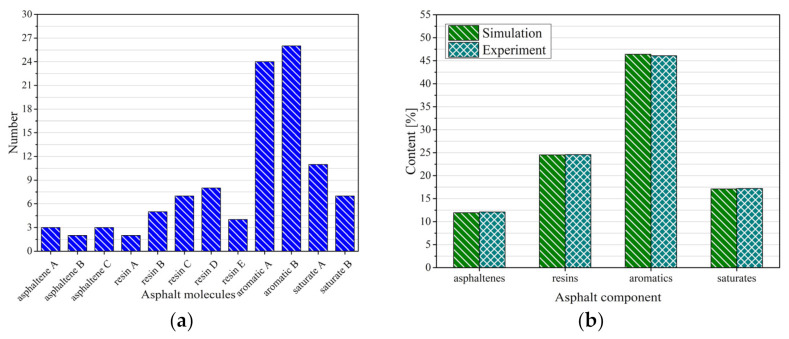
Number of molecules of the asphalt model (**a**) and the ratio of the model to the experiment in terms of four components (**b**).

In order to make the molecular model as close as possible to the asphalt sample, the four-component content of the base asphalt sample was first measured (ASTM D4124), and then the ratio of the number of various molecules was calculated based on the four-component measurements and the molecular masses of various asphalt molecules to make the four-component content of the model as close as possible to the four-component content of the asphalt. In this study, the number of various molecules of the base asphalt model and the four components of the sample are shown in Figure 4.

**Figure 5:**
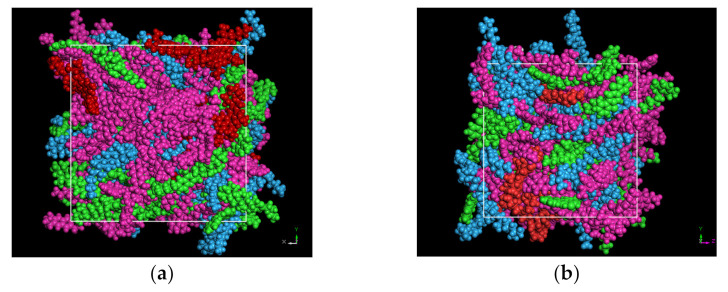

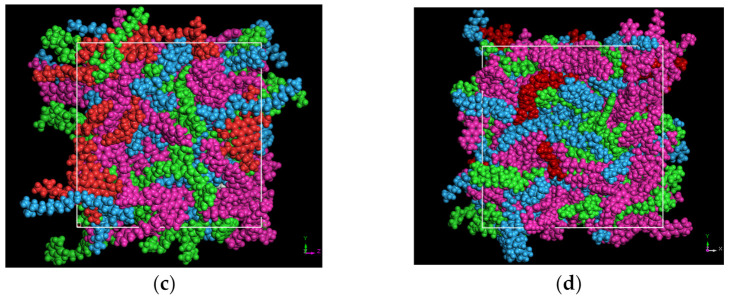
Molecular models of virgin asphalt (**a**), RPE-modified asphalt (**b**), CNT/RPE-modified asphalt (**c**) and the morphology of virgin asphalt after initial relaxation (**d**) (red indicates asphaltenes, green indicates resins, pink indicates aromatics and blue indicates saturates).

Based on the above, the molecular models of three systems—base asphalt, RPE-modified asphalt and CNT/RPE-modified asphalt—were constructed in the amorphous cell (AC) module of Materials Studio (MS) software, as shown in Figure 5.

**Figure 6:**
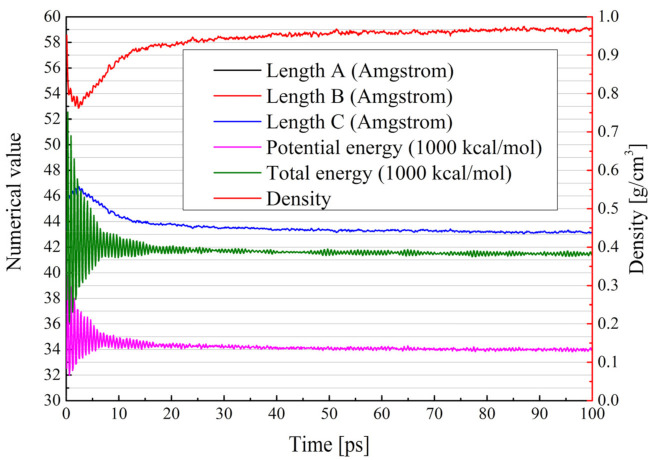
Variation of system parameters during molecular dynamics simulation.

All molecular dynamics simulations in this study were performed in Materials Studio (MS), the mapping of asphalt molecules was performed in the visualizer package and the construction of various asphalt systems was performed in the amorphous cell (AC) package. After obtaining the asphalt systems, they had to be optimized structurally. The first step was the geometry optimization of 100,000, and then each asphalt molecule was annealed at a temperature range of 300 to 500 K, with a cycle number of 5 and a temperature interval of 20 K. The annealing process was carried out in NPT with an air pressure of 1.01 × 10^−4^ Gpa, which was designed to simulate the real situation. Subsequently, a 50 ps dynamic simulation was performed under the NVE ensemble synthesis with the aim of the initial relaxation of each asphalt system. It has to be mentioned that the initial relaxation temperature was set to 400 K, as relaxation temperatures that are too low can lead to highly unbalanced asphalt systems. Finally, molecular dynamics simulations were per-formed for each asphalt system at 100 ps, in the NPT ensemble, at 298 K, with a total step size of 100,000 and one model frame output every 1000 steps. In other words, after the molecular dynamic simulation process, a 100-frame trajectory trace is the output, and the later calculations of various microscopic parameters are calculated in this trajectory file. The trends of the system parameter changes during the molecular dynamics simulation are shown in Figure 6. It can be observed that after the simulation proceeded to 30 ps, the asphalt had basically equilibrated.

The morphology of the virgin asphalt after the initial relaxation is shown in Figure 5d. It can be seen that after the initial relaxation, the asphaltene was in the center of the system, and the aromatics and saturates were surrounded by the asphaltene. This state supports the colloidal theory of asphalt and proves that the structure optimization process of this study is effective.

The force field used in molecular dynamics simulations is COMPASS, which has been adopted in many studies [33] and has proven to be suitable for calculating various properties and specifications of asphalt materials. The functional expression for the COMPASS force field is shown in Equation (1):

(1) $$\begin{array}{l} E_{total}=\sum_{bond}\left[K_{b2}{\left(b-b_{0}\right)}^{2}+K_{b3}{\left(b-b_{0}\right)}^{3}+K_{b4}{\left(b-b_{0}\right)}^{4}\right]\\ +\sum_{angle}\left[K_{a2}{\left(\theta -\theta_{0}\right)}^{2}+K_{a3}{\left(\theta -\theta_{0}\right)}^{3}+K_{a4}{\left(\theta -\theta_{0}\right)}^{4}\right]\\ +\sum_{torsion}\left[K_{t1}(1-\cos\phi )+K_{t2}(1-\cos2\phi )+K_{t3}(1-\cos3\phi )\right]\\ +\sum_{OOPA}K_{\chi }(\chi -\chi_{O})+\sum_{bond/bond}K_{bb}(b-b_{O})(b’-b{’}_{O})\\ +\sum_{bond/angle}K_{ba}(b-b_{O})(\theta -\theta_{0})\\ +\sum_{angle/angle}K_{aa}(\theta -\theta_{0})(\theta ’-\theta {’}_{0})\\ +\sum_{bond/torsion}(b-b_{O})\left[K_{bt1}(1-\cos\phi )+K_{bt2}(1-\cos2\phi )+K_{bt3}(1-\cos3\phi )\right]\\ +\sum_{angle/torsion}(\theta -\theta_{0})\left[K_{at1}(1-\cos\phi )+K_{bt2}(1-\cos2\phi )+K_{bt3}(1-\cos3\phi )\right]\\ +\sum_{angle/torsion/angle}k(\theta -\theta_{0})(\theta ’-\theta {’}_{0})(\phi -\phi_{O})\\ +\sum_{nonbond}\left\{\varepsilon_{ij}\left[2{\left(\frac{r_{ij}^{O}}{r_{ij}}\right)}^{9}-3{\left(\frac{r_{ij}^{O}}{r_{ij}}\right)}^{6}\right]+\frac{q_{i}q_{j}}{4\pi \varepsilon_{0}r_{ij}}\right\} \end{array}$$

The meanings of the various parameters in the above equation can be found in the literature [34,35].

### 2.4. Model Verification

The virgin asphalt model in this study was developed based on the four-component content of the asphalt samples, so the model’s four-component content was generally consistent with the sample values (Figure 4b). However, the agreement of the component content alone is not sufficient to conclude the reasonableness of the asphalt model. In this study, the model element content test (ASTM D7455), radial distribution function and glass transition temperature were calculated to verify the reasonableness of the asphalt model.

The contents of various elements in the asphalt samples and models are shown in Table 2. It can be seen that the asphalt had the highest content of C followed by H, which is in agreement with previous studies [36], indicating that asphalt is an organic mixture. The content of N, S and O was very low; however, they have a great influence on the properties of asphalt and are the source of asphalt polarity. The elemental content in the model is highly consistent with the sample values and although there are no impurities in the asphalt model, this difference is acceptable.

**Figure 7:**
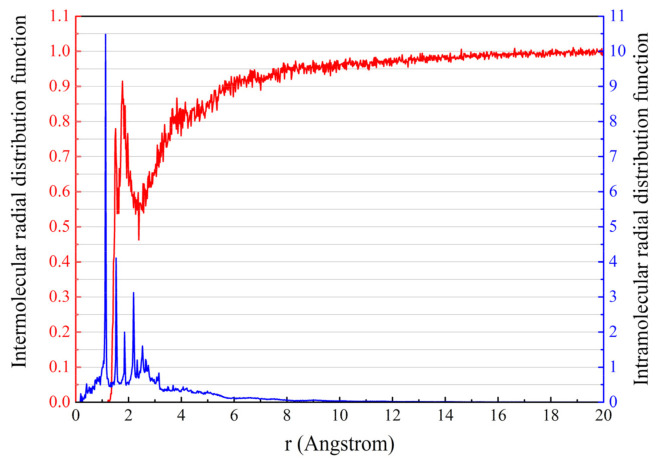
Results of the radial distribution function of the virgin asphalt model.

The intermolecular and intramolecular radial distribution functions of the virgin asphalt samples are shown in Figure 7. In Figure 7, it can be observed that the intramolecular radial distribution function shows multiple peaks when r < 4 Å and then gradually plateaus, which indicates that the virgin asphalt model is a remotely disordered and proximally ordered non-crystalline structure, a result that is consistent with the consensus. For the intermolecular radial distribution function curve, the radial distribution function gradually tends to 1 after r > 5 Å. This phenomenon indicates that the interactions in the virgin asphalt model are mainly concentrated in the range of 0–5 Å. Usually, the hydrogen bonds are in the range of 2.6–3.1 Å, whereas the van der Waals forces are in the range of 3.1–5.0 Å [37]. That is, the types of interactions in the virgin asphalt model are mainly hydrogen bonds and van der Waals forces, which are consistent with the actual asphalt.

**Figure 8:**
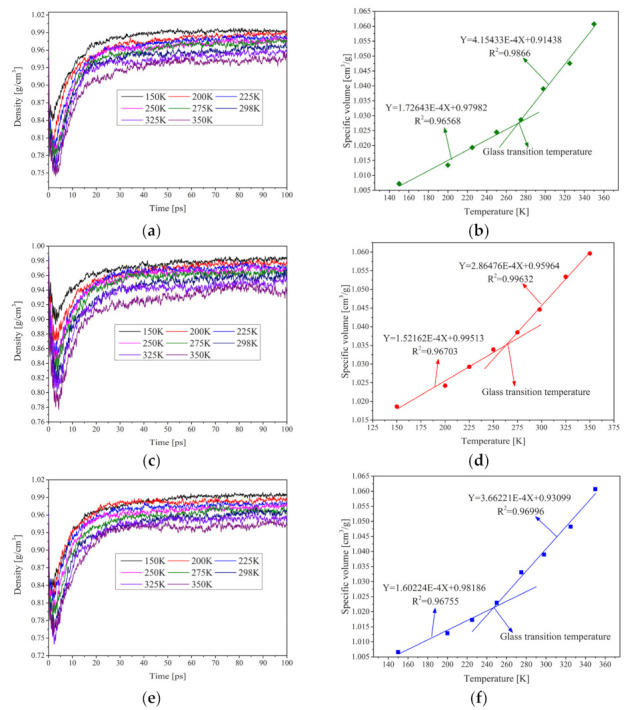
Density (**a**) and glass transition temperature (**b**) of virgin asphalt at different temperatures, density (**c**) and glass transition temperature (**d**) of RPE-modified asphalt at different temperatures, density (**e**) and glass transition temperature (**f**) of CNT-RPE-modified asphalt at different temperatures.

In addition, the glass transition temperatures of three asphalt models were calculated in this study to further validate the reasonableness of the models. A linear plot of the specific volume of different asphalt models versus different temperatures (150, 200, 225, 250, 275, 298, 325 and 350 K) was plotted, and the temperature corresponding to the abrupt change in slope was taken as the glass transition temperature of the model [25], and the results are shown in Figure 8. It can be seen that the density of the system gradually decreases with increasing temperature for virgin asphalt, RPE-modified asphalt and CNT-RPE-modified asphalt, a result that is consistent with the properties of asphalt materials [25]. In all the density time curves, the density remained essentially constant after 40 ps. In this study, the average density of the system was calculated by taking the density values between 70 and 100 ps and taking the inverse to obtain the specific volume of the system at different temperatures and fitting it linearly to finally obtain the glass transition temperature of different asphalt systems [38,39]. It can be seen that the coefficients of de-termination of the linear fits of specific volume and temperature are all greater than 0.96, which indicates that the linear relationship between temperature and specific volume is obvious, and the glass transition temperature of asphalt can be characterized at the change of slope.

The glass transition temperature of the virgin asphalt model is 272.38 K. Usually, the glass transition temperature of virgin asphalt is lower than 273.15 K [25], which is consistent with the calculated results. The glass transition temperature of the RPE-modified asphalt model is 264.23 K, which is significantly lower than that of the virgin asphalt model, which is consistent with previous studies [4], because RPE has an enhanced effect on the low-temperature properties of asphalt. Thus, the glass transition temperature of the RPE-modified asphalt model should be lower than that of the virgin asphalt model. In addition, the glass transition temperature of the CNT/RPE-modified asphalt model was lower than that of the RPE-modified asphalt, at 246.95 K, implying that the low-temperature properties of the CNT/RPE-modified asphalt were better than those of the RPE-modified asphalt, and this was proven by subsequent experiments.

In summary, based on the four-component content, elemental content, radial distribution function and glass transition temperature results, the virgin asphalt, RPE-modified asphalt model and CNT/RPE-modified asphalt models constructed in this study are rea-sonable.

### 2.5. Simulation Index

The binding energy, diffusion coefficient and relative concentration distribution of asphalt models were calculated as follows.

The binding energy is the energy consumed to separate a system that is joined together into two parts. The total energy of the system and the energy of separating the two parts are calculated separately, and the energy difference is the binding energy. If the binding energy is positive, it means that the two systems absorb each other. However, if the binding energy is negative, it means that the two systems hold each other. The larger the absolute value of the binding energy, the stronger the interaction between the two systems and the more stable they are [40]. The last frame of the dynamics simulation trajectory was chosen to calculate the binding energy between the different components of the molecular system, and the binding energy was calculated as shown in Equation (2):

(2) $$E_{Binding(asphalt/RPE)}=E_{asphalt}+E_{RPE}-E_{RPE/asphalt}$$

where, *E_Binding(asphalt/RPE)_* is the binding energy between one molecule of asphalt and RPE, *E_asphalt_* is the energy of one molecule of asphalt, *E_RPE_* is the energy of PE molecule and *E_asphalt/RPE_* is the total energy of one molecule of asphalt and PE molecule when they are together.

The diffusion coefficient characterizes the rate of motion of the molecules and is obtained from the MSD as a function of time, which is equal to one-sixth of the slope of the MSD as a function of time when the simulation time is long enough, as described by Ein-stein’s diffusion law. However, the literature [41,42] shows that for asphalt, the full result of MSD(t) cannot be used to calculate the diffusion coefficient, but rather the first small part should be used. Moreover, the diffusion coefficient should be calculated in a logarithm. This is performed as shown in Equation (3):

(3) $$Diffusion\;coefficients=\underset{t\to \infty }{lim}{\frac{\left|r(t)-r(0)\right|}{6t}}^{2}=\underset{t\to \infty }{lim}\frac{MSD(t)}{6t}=\frac{m}{6}$$

where, *r(t)* denotes the position of the molecule at the moment t and m denotes the slope of the *MSD(t)* function.

The relative concentration distribution can be obtained using the analysis function of the Forcite package. It is worth mentioning that the raw curve was smoothed to obtain a better regularity, and the width was chosen to be 3.0 bins.

## 3. Results and Discussion

### 3.1. Effect of CNT on the Road Properties of Modified Asphalt

**Figure 9:**
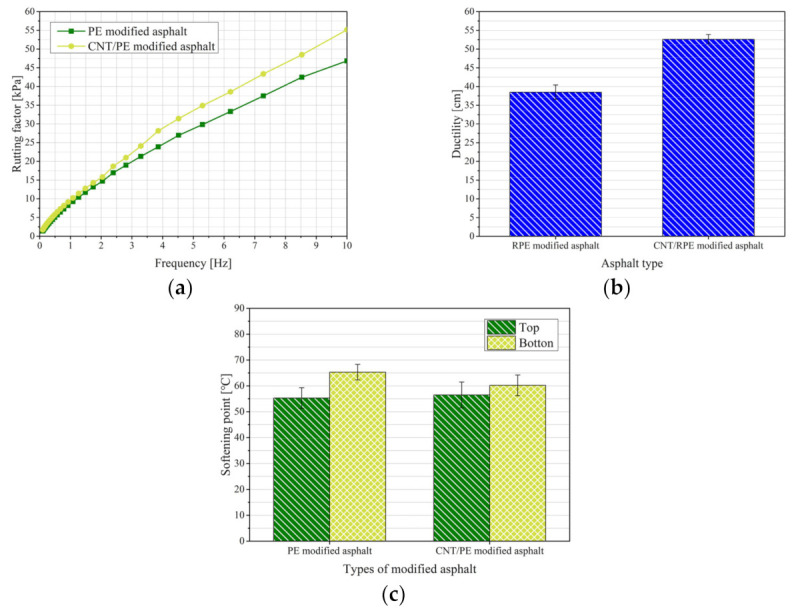
Properties of RPE-modified asphalt and CNT/RPE-modified asphalt.

The road properties of RPE-modified asphalt and CNT/RPE-modified asphalt are shown in Figure 9. In this study, the ductility of asphalt was characterized by means of the rutting factor. The rutting factor (Figure 9a) of RPE-modified asphalt increases with increasing frequency, which indicates that the anti-rutting property of RPE-modified asphalt is positively correlated with frequency. The rutting factor of CNT/RPE-modified asphalt is always greater than that of RPE-modified asphalt relative to RPE-modified asphalt, and the difference between the two increases with increasing frequency. This phenomenon indicates that the rutting resistance of RPE-modified asphalt is improved after the addition of CNT.

The low-temperature properties of asphalt were characterized by its ductility at 10 °C. The higher the ductility, the better the low-temperature properties. The ductility of RPE-modified asphalt and CNT/RPE-modified asphalt is shown in Figure 9b. It can be seen that the ductility of RPE-modified asphalt (38.5 cm) was higher than that of virgin asphalt (31.2 cm), which indicates that RPE has an enhanced effect on the low-temperature properties of asphalt, but the increase is not significant. However, the ductility of CNT/RPE-modified asphalt was 52.6 cm, which was significantly higher than that of RPE-modified asphalt, indicating that CNT significantly improved the low-temperature properties of asphalt and CNT/RPE-modified asphalt has good low-temperature properties.

If the softening point difference between the top and bottom part of the asphalt samples in the test tube experiment is less than 5 °C, the asphalt samples can be considered to have good storage stability, and if it is greater than 5 °C, the asphalt samples are considered to have poor storage stability [43]. The results of the storage stability experiments for RPE-modified asphalt and CNT/RPE-modified asphalt are shown in Figure 9c. It can be seen that the difference in softening point between the top (55.3 °C) and bottom (65.3 °C) of RPE-modified asphalt is 10 °C, which predicts that the storage stability of RPE-modified asphalt is poor, and that phase separation of RPE-modified asphalt can easily occur during high-temperature storage, which may be due to the smooth surface of RPE and its low adhesion to asphalt. However, the difference in softening point between the top (56.5 °C) and bottom (60.2 °C) of CNT/RPE-modified asphalt is 3.7 °C, which predicts a good storage stability of CNT/RPE-modified asphalt.

### 3.2. Interaction Stability and Morphology of RPE-Modified Asphalt

The binding energies between various molecules in the virgin asphalt, RPE-modified asphalt and CNT/RPE-modified asphalt systems are shown in Table 3, Table 4 and Table 5.

In Table 3, it can be observed that the binding energies between asphaltenes and other components of asphalt are ranked as follows: aromatics (2700.93 kcal/mol) > saturates (701.76 kcal/mol) > resins (232.26 kcal/mol). The binding energies are all greater than 0. This indicates that the interaction of asphaltenes with other components are attractive, and asphaltenes are the most stable with aromatics, the least stable with resins, and that the binding with saturates occurs between aromatics and resins. The colloid theory of asphalt suggests that asphalt is a dispersion system consisting of asphaltenes and adsorbed resins as dispersions, distributed in aromatics and saturates. In other words, the key to the formation of a stable colloidal system of asphaltenes is the interaction between asphaltenes and lighter components (aromatic and saturates), whereas resins are a transitional substance. Therefore, the binding energy of asphaltenes with saturates and aromatics is much larger than that of resins.

In Table 4, it can be observed that in the RPE-modified asphalt system, the binding energy of asphaltenes with each component of the asphalt was significantly lower than that of the virgin asphalt, but the size trend did not change. This means that the introduction of RPE did not change the colloidal structure of the asphalt but weakened the interaction within the asphalt. In addition, the magnitude of the binding energy of PE with the four components of asphalt was ranked as follows: aromatics (225.23 kcal/mol) > saturates (118.01 kcal/mol) > resins (−35.28 kcal/mol) > asphaltenes (−423.82 kcal/mol). It is worth noting that the binding energy of PE with asphaltenes and resins is negative, which indicates that PE and asphaltenes and resins are repulsive to each other. The binding energy of PE with aromatics and saturates is positive, which means that PE and these components will be attracted to each other, that is, when RPE is added to the base asphalt, RPE will absorb a portion of the light components. However, the binding energy between PE and light components is much lower than that between asphaltene and light components, which means that RPE can adsorb few light components and RPE cannot form a three-dimensional network structure like styrene–butadiene–styrene (SBS). This has been demonstrated in previous studies, in which PE tended to form spherical “island structures” in the base asphalt [44]. The binding energy results can explain the poor storage stability of RPE-modified asphalt, because the interaction between PE and lighter components is much weaker than asphaltenes, and it is mutually exclusive with asphaltenes and resins, and the molecular structure, polarity and thermodynamic properties of PE are very different from those of asphalt, so the compatibility between PE and asphalt is very low and it is easy to separate them under special circumstances.

Table 5 shows the results of binding energy calculations between various molecules in CNT/RPE-modified asphalt. The order of binding energy between asphaltenes and other components remains the same, which indicates that the asphalt phase in CNT/RPE-modified asphalt is still a colloidal system. However, the binding energy between asphaltenes and other components in the CNT/RPE-modified asphalt system was larger compared to that of RPE-modified asphalt, which indicates that CNTs can enhance the stability of the asphalt phase in the system. In addition, the binding energy of PE with the four components also changed significantly. The binding energy of PE with asphaltenes decreased (−253.17 kcal/mol), indicating that the repulsion between PE and asphaltenes was reduced, which contributed to the stability of the modified asphalt system. The binding energy of PE with resins became positive (10.32 kcal/mol), which indicates that CNTs cause the relation between PE and resins to change from mutual repulsion to mutual attraction. In addition, the binding energy of PE with aromatics (562.84 kcal/mol) and saturates (326.16 kcal/mol) in CNT/RPE-modified asphalt was significantly higher than that of RPE-modified asphalt, which indicates that CNTs promote the adsorption of lighter components by PE. The above results can explain the experimental results shown in Figure 8, because CNTs not only weaken the repulsive effect with RPE with asphaltenes and resins, they also enhance the interaction of RPE with lighter components. This process induces further dissolution of RPE in the base asphalt, and the excellent properties of RPE are transferred to the whole system, making the high-temperature stability and low-temperature properties of CNT/RPE-modified asphalt higher than that of RPE-modified asphalt. In addition, the storage stability of CNT/RPE-modified asphalt is better, due to the attachment of more lightweight components outside of RPE, which are equivalent to the transition region and increase the compatibility of RPE with the asphalt matrix.

**Figure 10:**
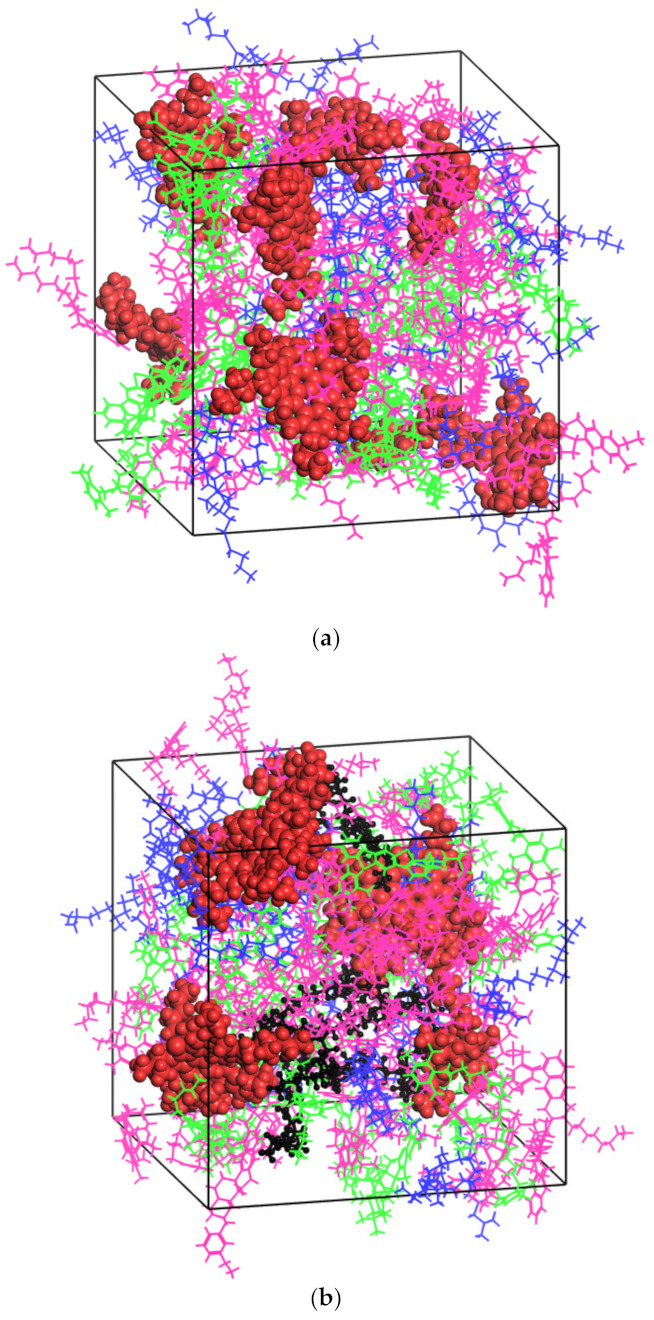

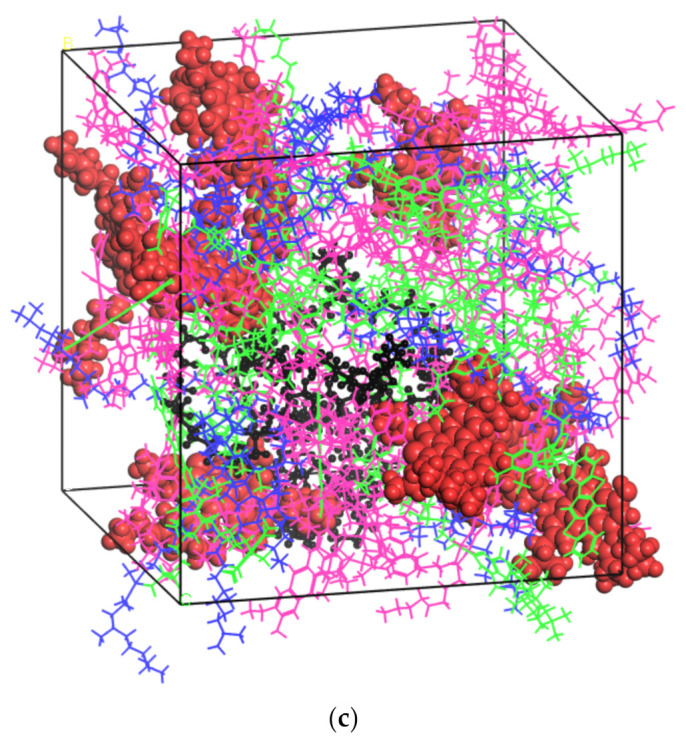
Interaction morphology of virgin asphalt (**a**), RPE-modified asphalt (**b**) and CNT/RPE-modified asphalt (**c**) (red for asphaltenes, green for resins, pink for aromatics, blue for saturates and black for PE).

The system morphology at kinetic equilibrium for virgin asphalt, RPE-modified asphalt and CNT/RPE-modified asphalt is shown in Figure 10. In the virgin asphalt (Figure 10a), the asphaltenes are uniformly distributed throughout the system. According to the colloid theory of asphalt, asphaltenes are the main source of system elasticity, adsorbing resins to form colloids which are distributed among the aromatics and saturates. The uniform asphaltene distribution implies that the colloidal system of virgin asphalt is stable. In RPE-modified asphalt (Figure 10b), the asphaltenes are more cohesive and the PE molecular chains are interspersed in the asphaltenes in strips. According to the binding energy results, the asphaltenes and PE repel each other. The PE molecular chains are interspersed in the asphaltenes, which means that the RPE-modified asphalt system is not stable. In addition, the PE molecular chains are in strips, which means that the contact surface between PE and asphaltenes is larger, which aggravates the instability of RPE-modified asphalt. In contrast, in CNT/RPE-modified asphalt, the asphaltene has significantly more space for distribution, which facilitates the stability of the system. In addition, the PE molecular chains are distributed in clusters, and this state reduces the contact area between PE and asphaltene, which weakens the repulsion reaction. In other words, CNTs enhance the stability of RPE-modified asphalt, which is consistent with the binding energy results.

### 3.3. Effect of CNT on Molecular Diffusion of RPE-Modified Asphalt

**Figure 11:**
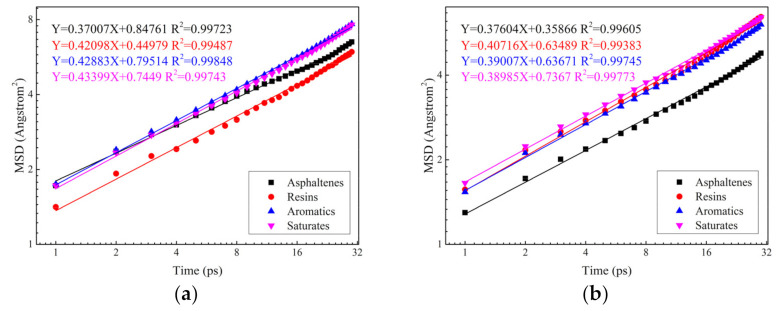

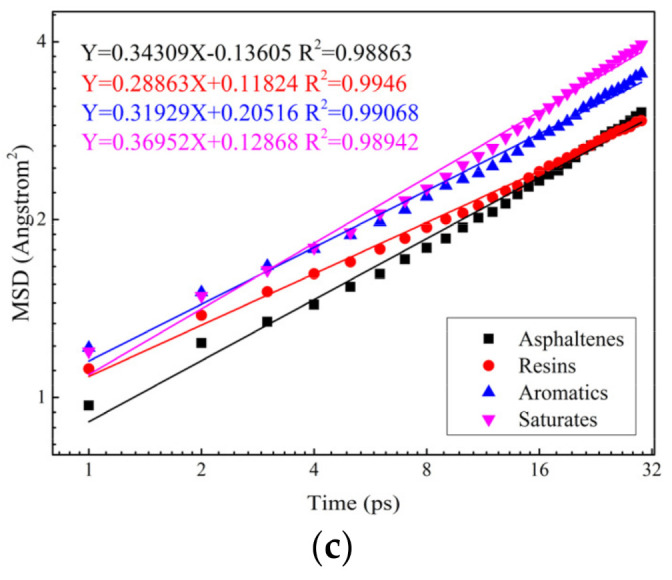
MSD results for virgin asphalt (**a**), RPE-modified asphalt (**b**) and CNT/RPE-modified asphalt (**c**).

**Figure 12:**
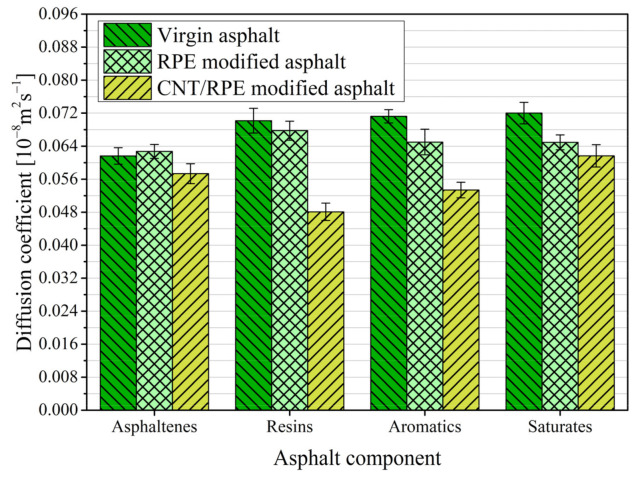
Results of diffusion coefficients for three asphalts.

The four-component MSD results for virgin asphalt, RPE-modified asphalt and CNT/RPE-modified asphalt are shown in Figure 11. It can be seen that the coefficient of determination of MSD versus time is greater than 0.98 in all three asphalts, which indicates that the linear relationship between MSD and time is obvious. In other words, the diffusion coefficient, which can be calculated using the MSD-t fitting slope, is able to characterize the diffusion behavior of the component during the kinetic simulation. The results of the four-component diffusion coefficients for virgin asphalt, RPE-modified asphalt and CNT/RPE-modified asphalt in this study are shown in Figure 12.

As shown in Figure 12, the order of the diffusion coefficients of the four components in virgin asphalt, from largest to smallest, is: saturates (0.072 × 10^−8^ m^2^s^−1^) > aromatics (0.071 × 10^−8^ m^2^s^−1^) > resins (0.070 × 10^−8^ m^2^s^−1^) > asphaltenes (0.062 × 10^−8^ m^2^s^−1^). This result indicates that the diffusion of the components in the virgin asphaltenes is significantly related to the molecular mass: the larger the molecular weight, the slower the molecular diffusion, and the smaller the molecular weight, the faster the molecular diffusion. This is in agreement with the results of previous studies [45]. However, in RPE-modified asphalt, the diffusion coefficients of the four components of asphaltenes were (ranked from largest to smallest): (0.067 × 10^−8^ m^2^s^−1^) > aromatics (0.065 × 10^−8^ m^2^s^−1^) > saturates (0.0649 × 10^−8^ m^2^s^−1^) > asphaltenes (0.063 × 10^−8^ m^2^s^−1^), which indicates that the interaction between RPE and asphalt molecules has an important effect on the diffusion of asphalt molecules. The diffusion coefficient of asphaltenes in RPE-modified asphalt is larger than that of virgin asphalt, which is due to the obvious repulsive effect of RPE on asphaltenes, and this repulsive effect promotes the movement of asphaltenes. The diffusion coefficient of aromatics and saturates in RPE-modified asphalt is significantly smaller than that of virgin asphalt, which is due to the mutual attraction between RPE and these components, and this attraction binds the diffusion of lightweight components.

In addition, the diffusion coefficients of the four components in CNT/RPE-modified asphalt were ranked as follows: saturates (0.062 × 10^−8^ m^2^s^−1^) > asphaltenes (0.057 × 10^−8^ m^2^s^−1^) > aromatics (0.053 × 10^−8^ m^2^s^−1^) > resins (0.048 × 10^−8^ m^2^s^−1^). This is because the addition of CNTs enhances the binding energy of RPE to resins, aromatics and saturates, and the attraction of RPE to these components increases, leading to a reduction in their diffusion coefficients. However, the RPE remains repulsive with asphaltenes. Under the effect of RPE repulsion and the attractive effect of RPE with the remaining components, the diffusion coefficients of asphaltenes in our study were larger than those of aromatics and saturates, although the molecular weight of asphaltenes was the largest.

### 3.4. Molecular Structure Analysis of Different Asphalt Systems

**Figure 13:**
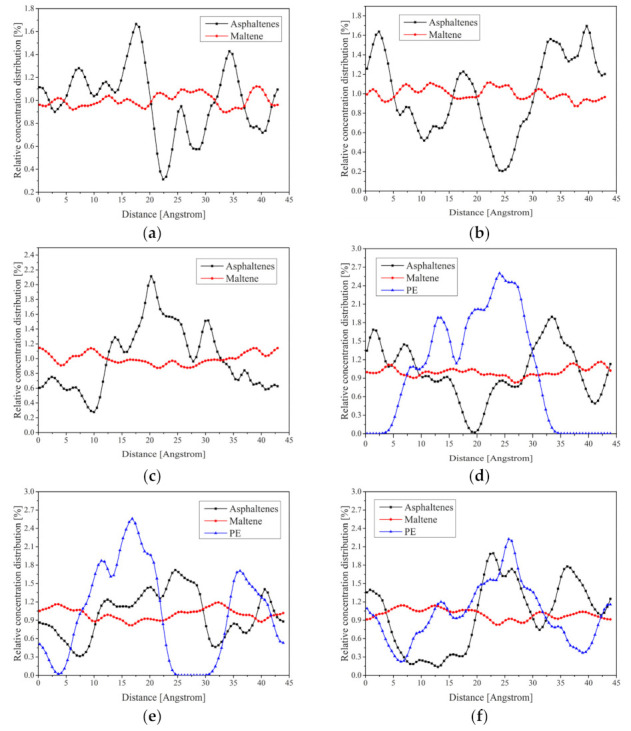

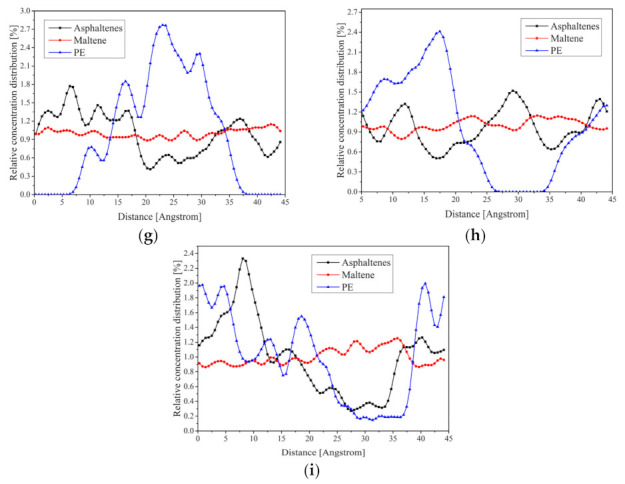
Concentration distribution of virgin asphalt, RPE-modified asphalt and CNT/RPE-modified asphalt in X, Y and Z directions. (**a**) Virgin asphalt-X, (**b**) Virgin asphalt-Y, (**c**) Virgin asphalt-Z, (**d**) RPE-modified asphalt-X, (**e**) RPE-modified asphalt-X, (**f**) RPE-modified asphalt-X, (**g**) CNT/RPE-modified asphalt-X, (**h**) CNT/RPE-modified asphalt-Y, (**i**) CNT/RPE-modified asphalt-Z.

The concentration distributions of virgin asphalt, RPE-modified asphalt and CNT/RPE-modified asphalt in the X, Y and Z directions are shown in Figure 13. It can be observed that in the virgin asphalt system (Figure 13a–c), there are multiple peaks of asphaltenes in either direction, which indicates a decisive influence of asphaltenes on the properties of asphalt materials. The asphaltene peaks are distributed throughout the region, indicating that the asphaltene in the virgin asphalt is relatively uniformly dispersed throughout the system, and the virgin asphalt system is a sol-gel type colloid. As for maltene, which consists of resins, aromatics and saturates, it is very uniformly distributed in three directions without obvious peaks, which indicates that maltene acts as a dispersion to dissolve asphaltenes. The above conclusion is consistent with the colloid theory.

For the RPE-modified asphalt (Figure 13d–f), the maltenes remain uniformly distributed. They are also the dissolved substances of the system, acting as dissolved dispersions. However, the number of asphaltene peaks is significantly less than in the virgin asphalt, and the asphaltenes peaks have a tendency to move toward the middle, a result consistent with the system’s morphological results (Figure 10b). In addition, there is also a distinct peak in RPE with a higher peak than asphaltenes, which indicates a concentrated distribution in RPE-modified asphalt. Observing the position of the RPE peak, it was found that in the X direction, the peak of RPE appeared to coincide with the trough of asphaltenes, which reflected the repulsive effect of RPE and asphaltenes. However, in the Y and Z directions, there is an obvious overlap between the RPE peaks and the asphaltene peaks, a phenomenon that would lead to the inability of RPE to react with enough lighter components, thus leading to phase separation.

As for the CNT/RPE-modified asphalt (Figure 13g–i), the distribution of the asphaltenes peak is wider than that of the RPE-modified asphalt, which indicates that CNTs make the distribution of asphaltenes more uniform, which is beneficial to the stability of the system. RPE still has only one distinct peak, but compared to the RPE-modified asphalt, the difference between the RPE peak and the asphaltenes peak is larger, which indicates that the addition of CNTs makes the RPE more aggregated. The above results are consistent with the morphological results of CNT/RPE-modified asphalt (Figure 10c). Notably, the RPE peak appears at the trough of the asphaltenes, which means that the distance between the RPE peak and the asphaltene peak increases, and this phenomenon is beneficial to the stability of the modified asphalt system. The RPE of principal asphaltenes can adsorb more light components, which enhances the compatibility of RPE with asphalt and also leads to the improved storage stability of CNT/RPE-modified asphalt.

## 4. Conclusions

In order to improve the storage stability and low-temperature properties of RPE-modified asphalt, CNTs were used as a reinforcing agent in this study, and molecular dynamics simulation was used to explore the reinforcing mechanism of CNTs on RPE-modified asphalt. The following conclusions were obtained.

- CNTs effectively enhance the high-temperature rheological properties, low-temperature cracking resistance and storage stability of RPE-modified asphalt.
- The glass transition temperature, elemental content and four-component content justify the molecular models of virgin asphalt, RPE-modified asphalt and CNT/RPE-modified asphalt.
- The enhancement mechanism of CNTs on RPE is that CNTs not only weakened the interaction between asphaltene and light components and reduced the repulsion effect between RPE and asphaltene (resins), but also enhanced the adsorption of RPE and light components, which meant that RPE adsorbed more light components and facilitated the compatibility between RPE and base asphalt.
- CNTs make the asphaltene distribution more uniform and increase the distance be-tween RPE and asphaltene, which further improves the storage stability of the modified asphalt.

## Figures and Tables

**Table 1:** Basic properties of base asphalt.

Projects		Units	Test Results	Technical Requirements	Test Method
Ductility (10 °C)		cm	31.2	>15	ASTM D 113
Ductility (15 °C)		cm	152.8	>100	ASTM D 113
Softening point		°C	49.4	>46	ASTM D36
Viscosity, 135 °C		Pa · s	0.572	-	ASTM D4402
Penetration (25 °C)		0.1 mm	65.2	60–80	ASTM D 05
After RTFOT	Ductility (10 °C)	cm	12	>6	ASTM D 113
	Ductility (15 °C)	cm	115.3	>15	ASTM D 113

**Table 2:** Comparison of elemental content of asphalt samples and models.

Element (wt%)	C	H	N	S	O	The Rest
Sample	86.563	9.875	0.327	1.426	1.784	0.025
Model	87.228	10.667	0.359	1.506	0.240	0

**Table 3:** Comparison of elemental content of asphalt samples and models.

Projects	Binding Energy	Bond Energy	Non-Bond Energy		
			van der Waals	Long-Range Correction	Electrostatic
Asphaltenes-Resins	232.26	0	238.54	5.3	0.99
Asphaltenes-Aromatics	2700.93	0	2685.69	10.52	4.72
Asphaltenes-Saturates	701.76	0	692.52	3.90	5.34

**Table 4:** Binding energy between components in RPE-modified asphalt.

Projects	Binding Energy	Bond Energy	Non-Bond Energy		
			van der Waals	Long-Range Correction	Electrostatic
Asphaltenes-Resins	132.67	0	125.73	3.82	3.12
Asphaltenes-Aromatics	1167.14	0	1137.05	15.92	14.17
Asphaltenes-Saturates	343.99	0	337.17	2.04	4.78
PE-Asphaltenes	−423.82	0	−414.74	−2.94	−6.14
PE-Resins	−35.28	0	−31.04	−2.18	−2.06
PE-Aromatics	225.23	0	219.63	5.58	0.02
PE-Saturates	118.01	0	114.91	2.81	0.29

**Table 5:** Binding energy between components in CNT/RPE-modified asphalt.

Projects	Binding Energy	Bond Energy	Non-Bond Energy		
			van der Waals	Long-Range Correction	Electrostatic
Asphaltenes-Resins	168.24	0	149.28	4.29	14.67
Asphaltenes-Aromatics	1392.02	0	1376.93	6.06	9.03
Asphaltenes-Saturates	528.31	0	521.03	8.35	−1.07
PE-Asphaltenes	−253.17	0	−241.23	−6.27	−5.67
PE-Resins	10.32	0	8.14	1.21	0.97
PE-Aromatics	562.84	0	553.98	5.94	2.92
PE-Saturates	326.16	0	306.28	3.92	15.96
